# A New Member of the Metal‐Porphyrin Frameworks Family: Structure, Physicochemical Properties, Hydrogen and Carbon Dioxide Adsorption

**DOI:** 10.1002/open.202300100

**Published:** 2023-11-09

**Authors:** Nikolas Király, Vladimír Zeleňák, Tomáš Zelenka, Miroslav Almáši, Juraj Kuchár

**Affiliations:** ^1^ Department of Inorganic Chemistry P. J. Šafárik University Moyzesova 11 041 01 Košice Slovak Republic; ^2^ Department of Chemistry University of Ostrava 30. Dubna 22 702 00 Ostrava Czech Republic

**Keywords:** carbon dioxide adsorption, hydrogen adsorption, isosteric heats, metal-organic frameworks, metal-porphyrin frameworks

## Abstract

A novel holmium‐based porous metal‐porphyrin framework, {(H_3_O^+^)[Ho(H_2_TPPS)]^−^ ⋅ 4H_2_O}_n_ (denoted as **UPJS‐17**), was synthesised by hydrothermal reaction. Structural analysis reveals, that **UPJS‐17** has a three‐dimensional open framework. The framework is negatively charged and the negative charge is compensated by hydronium cation. The compound showed no N_2_ adsorption but the Ar, CO_2_ and H_2_. From the argon adsorption, the surface area of ~150 m^2^ g^−1^ was determined. Carbon dioxide adsorption was measured at various temperatures (0, 10, 20, 30 and 40 °C) and the compound showed the highest adsorption capacity (at 0 °C) of 7.0 wt % of CO_2_. From the carbon dioxide adsorption isotherms the isosteric heat of 56,5 kJ mol^−1^ was determined. Hydrogen adsorption was studied at −196 °C with hydrogen uptake of 2.1 wt % at 1 bar.

## Graphical abstract

A novel metal‐porphyrin framework containing Ho^3+^ ions was prepared and fully characterized. Adsorption of hydrogen was studied at three different temperatures, and the adsorption of carbon dioxide was investigated at five different temperatures. Isosteric heats of carbon dioxide adsorption were calculated.

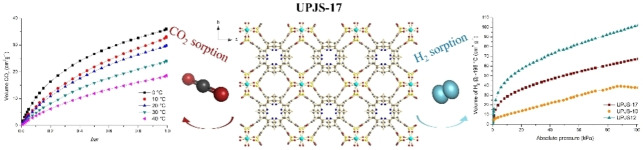

## Introduction

Metal‐porphyrin frameworks (MPFs), as a subclass of widely studied metal‐organic frameworks (MOFs), have been intensively developed as a promising new type of functional porous materials over the past decades. However, only in the last decade, chemically robust and permanently porous MPF materials have been achieved.[^1^, ^2^] The designability of their structures, tunability of pore sizes and modularity of properties by crystal engineering strategy[^3^, ^4^] as well as their permanent porosity afford them great potential for applications in gas storage and separation,[^5^, ^6^, ^7^] heterogeneous catalysis,[^8^, ^9^] magnetism,^[10]^ biomedical applications[^11^, ^12^] or sensing.^[13]^ Tetratopic and pentatopic square planar building blocks based on tetraarylporphyrins, such as 5,10,15,20‐tetrakis(4‐pyridyl)porphyrin (H_2_TPyP)^[14]^ and 5,10,15,20‐tetrakis(4‐carboxyphenyl)porphyrin (H_6_TCPP),^[15]^ have been used extensively as ligands in the synthesis of different MPFs with the almost all metal ions within the periodic table.[^14^, ^15^] Recently, a new MPF member with tetratopic 5,10,15,20‐tetrakis(4‐phenylphosphonic acid)porphyrin (H_8_TPPA) linker was published.^[16]^ However, the meso‐tetraphenylporphirin‐4,4′,4′′,4′′′‐tetrasulfonic acid as a ligand (H_6_TPPS), in combination with lanthanide metal ions, was relatively less investigated and found in the literature.^[17]^ In this contribution, we expand our previous research, and we report the preparation and properties of holmium‐based porous MPFs with H_6_TPPS, namely {(H_3_O^+^)[Ho(H_2_TPPS)]^−^ ⋅ 4H_2_O}_n_ (**UPJS‐17**).

## Results and Discussion

**Figure 1:**
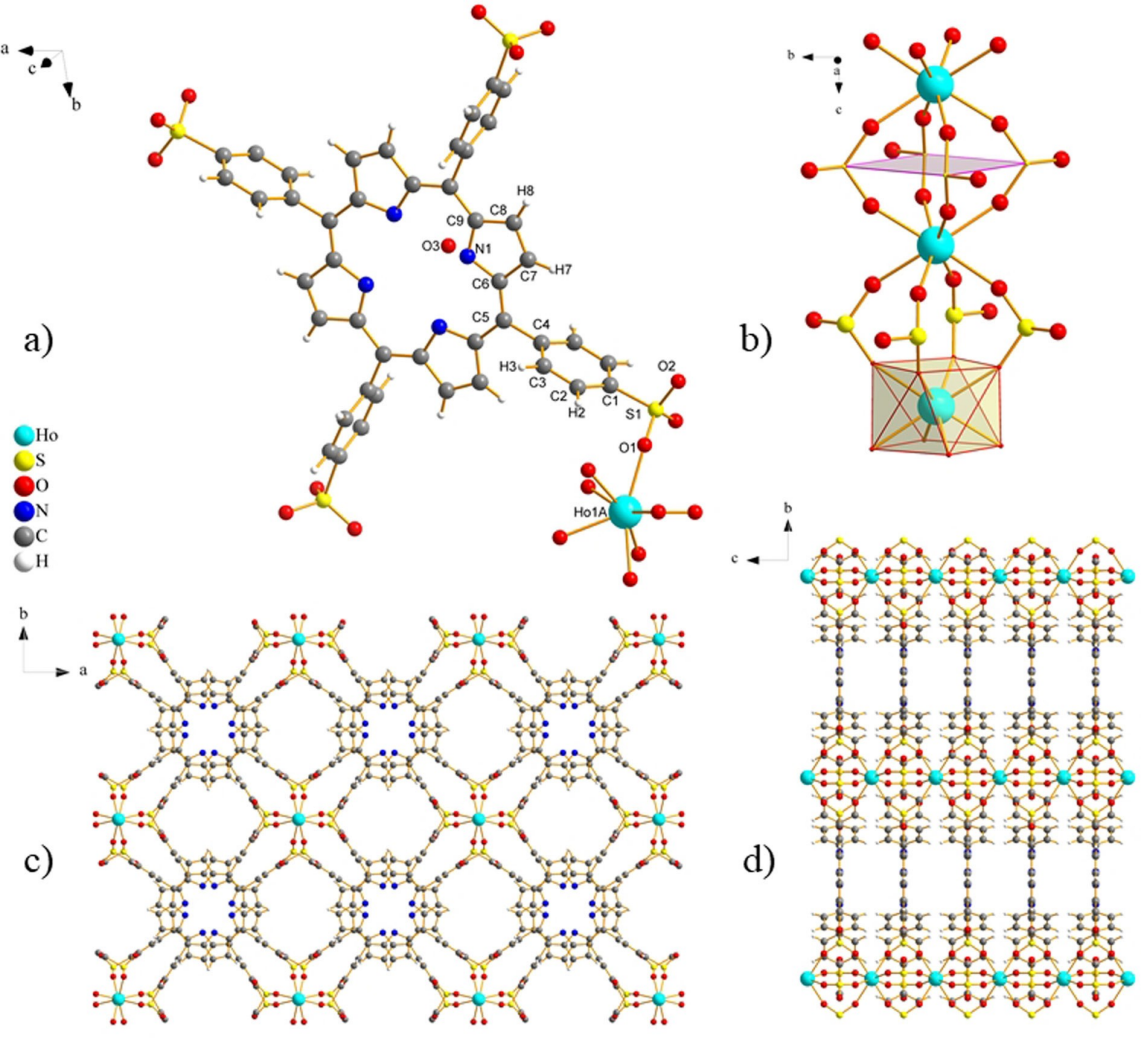
a) Coordination environment of **UPJS‐17**, with labelling of atoms in asymmetric unit, b) square planar SBU and deformed square antiprismatic coordination geometry of Ho(III) ions and view of ‐Ho‐(SO_3_)_4_‐Ho‐(SO_3_)_4_‐Ho‐ chains along the crystallographic *c* axis, c) 3D polymeric framework along the crystallographic *c* axis, d) 3D polymeric framework along the crystallographic *a* axis.

Dark green needle crystals of **UPJS‐17** were prepared by hydrothermal reaction of Ho(NO_3_)_3_ ⋅ 6H_2_O with the H_6_TPPS acid, in distilled water for 5 days (see Supporting Information for details). Single‐crystal X‐ray diffraction analysis revealed that **UPJS‐17** (Figure 1a) crystallizes in the space group P*4/mcc* (crystal data and details of XRD determination are summarized in Table S1 in the Supporting Information). The asymmetric unit of **UPJS‐17** contains C_1‐9_, H_2,3,7,8,_ N_1_, S_1_, O_1‐3_ and Ho1 A atoms (for the atom labels see Figure 1a). One H_2_TPPS^4−^ ligand bridges eight independent Ho(III) ions. In this coordination, the neighbouring Ho(III) ions are connected by deprotonated −SO_3_H groups, leading to the creation of the chains of −Ho‐(SO_3_)_4_‐Ho− dimers along the *c* crystallographic axis (Figure 1b). In such dimers the square planar secondary building units (SBUs) are present (see pink square Figure 1b). Each Ho(III) atom possess a deformed square antiprismatic coordination geometry. Furthermore, the interconnection of −Ho‐(SO_3_)_4_‐Ho− chains through H_2_TPPS^4−^ ligand leads to the formation of an open porous three‐dimensional negatively charged framework containing three mutually crossing channels propagating along all crystallographic axes (Figure 1c, d). The sizes of the channels are approximately 5.16×8.16 Å^2^ (along *c* axis) and 4.89×10.42 Å^2^ (along *a* and *b* axis) (Figure 1c, d). Moreover, in the centre of each cavity, hydronium H_3_O^+^ cations are located, which balance the negative charge of the framework. Moreover, four crystallization water molecules are present in the structural channels of the compound. The presence of hydronium cations and crystallization water molecules in the pores was confirmed by DRIFT, TG and elemental analyses (see Supporting Information). The total free pore volume in **UPJS‐17** evaluated by PLATON/SQUEEZE tool^[18]^ is 9 % (201 Å^3^). The topological analysis determined using the ToposPro program^[19]^ showed that **UPJS‐17** has 3 2,6‐c net topology using a standard representation of the covalent compounds, not imitating any known nature mineral topology.

However, if the cluster is considered as a single node representation, then the framework of the compound can be described as *fsc*‐net.

**Figure 2:**
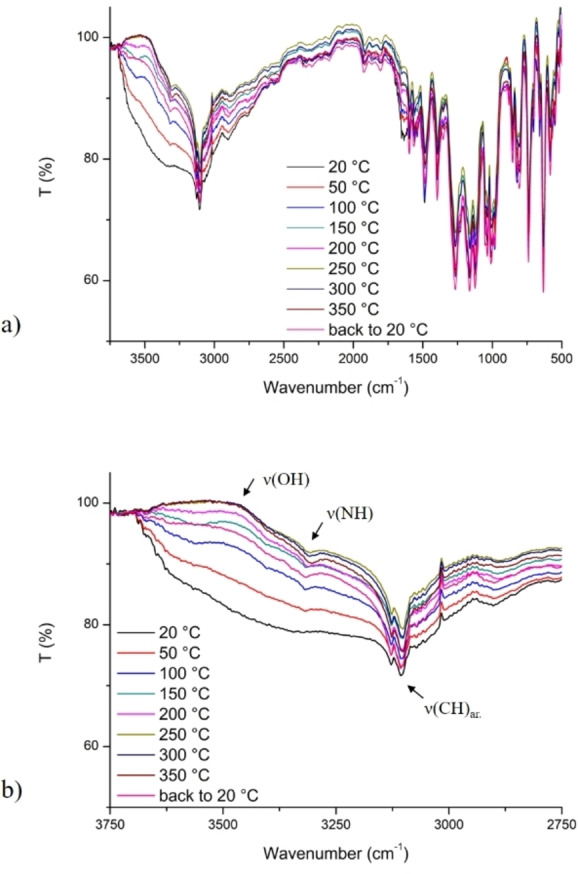
a) Full spectrum of in‐situ heating DRIFT spectra of **UPJS‐17** at selected temperatures (20, 50, 100, 150, 200, 250, 300 and 350 °C) under vacuum, b) area 3750–2750 cm^−1^, for clearer evidence of decreasing intensity of ν(OH) vibration.

The phase purity and the bulk composition of **UPJS‐17** were verified by powder X‐ray diffraction studies, which revealed that the XRD pattern calculated from SXRD and experimentally measured pattern of as‐synthetized sample are consistent (see Figure S1 in the Supporting Information). The infrared spectra (see Figure 2) confirmed the presence of H_2_TPPS^4−^ ligands in **UPJS‐17**, and DRIFT spectra measured during *in‐situ* heating of **UPJS‐17** confirmed its high thermal stability (from 20 °C to 350 °C). The presence of crystallization water molecules in **UPJS‐17** was confirmed by a broad absorption band centred at 3505 cm^−1^ which can be attributed to the O−H stretching vibrations. During the *in‐situ* heating of the sample, the DRIFT spectra in this region show a decrease in the intensity of this band due to removal of water molecules from the pores (Figure 2a). The presence of H_2_TPPS^4−^ ligand was confirmed by vibrations of pyrrole: ν(N−H) vibration at 3319 cm^−1^ (clearer evidence in the spectrum measured at 350 °C, Figure 2b) and also by stretching vibrations of aromatic C−H groups about 3095 cm^−1^, the stretching vibrations of aromatic C=C groups at around 1620 cm^−1^, stretching vibrations of C−N−C bond at 1481 cm^−1^, the asymmetric and symmetric stretching vibrations of ν(O−S−O) observed at 1167 and 1032 cm^−1^, respectively. Moreover, absorption bands of C−S stretching vibrations and deformation vibration δ(SO^3−^) at 731 cm^−1^ and 624 cm^−1^, respectively were observed.

To study the thermal stability of the framework and the solvatation, **UPJS‐17** was subjected to thermogravimetric analysis (TGA). TGA/DTA curve (see Figure S2 in the Supporting Information) exhibits a first weight loss of 7.8 % in the temperature range of 100–120 °C, corresponding to the loss of guest water molecules trapped in the pores (calc. 7.7 %). The dehydration is accompanied by endothermic effects on DTA curve. After the water release, the compound was thermally stable with a relative plateau observed on the TG curve from 120–400 °C. Above 400 °C continuous weight loss, corresponding to complete decomposition of the holmium‐porphyrin framework was observed (loss 71.95 %). During the thermolysis of the framework, three exothermic effects on DTA were observed, namely at the temperatures of 460 °C, 580 °C and 670 °C. The residual mass was 15.77 %, corresponding to Ho_2_O_3_ as a white powder (calc. 15.91 %). The knowledge gained from the thermal analysis and DRIFT spectra show on the robustness of the framework and the possibility of its activation for gas sorption experiments.

**Figure 3:**
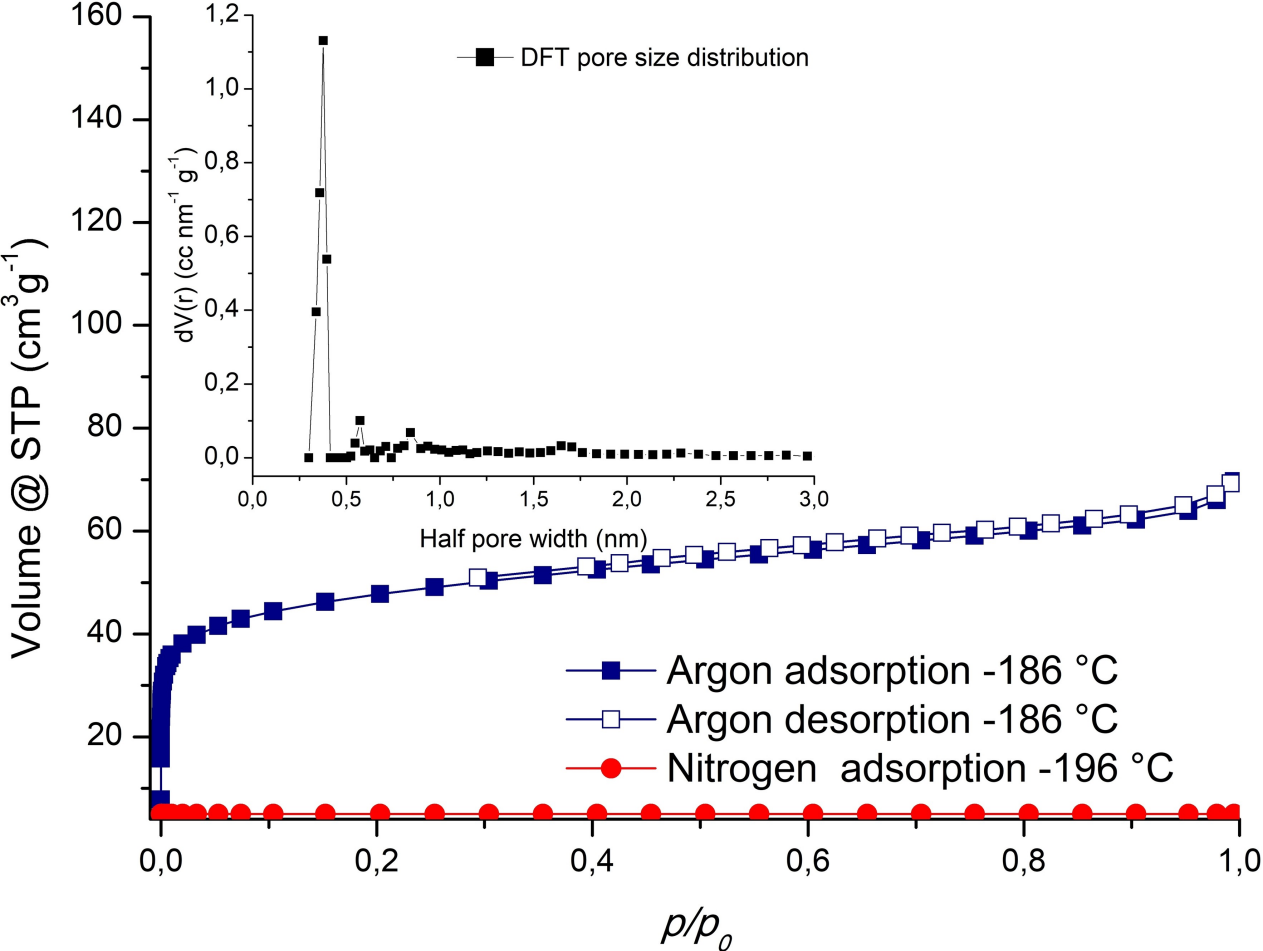
Argon adsorption/desorption isotherm measured at −186 °C (blue curve) and nitrogen adsorption measurements at −196 °C (red curve). Inset shows plot of the DFT pore size distribution (black curve).

To evaluate the permanent porosity and to study the gas sorption properties of **UPJS‐17**, adsorption of various gases (N_2_, Ar, CO_2_, H_2_) was performed on the sample after activation (activation process is described in the Supporting Information). Sample after activation and adsorption was stable, as confirmed by PXRD results, presented in Figure S1 in the Supporting Information (blue curve). As shown in Figure 3 (red curve), **UPJS‐17** adsorbs nearly no N_2_ at −196 °C, which can be presumably due to the small pore size precluding the access of nitrogen molecules into the pores. However, the adsorption of argon at −186 °C reveals that **UPJS‐17** exhibits an uptake of ~70 cm^3^ g^−1^ at 1 bar. The argon adsorption isotherm of type‐*I* was observed for **UPJS‐17**, with complete desorption of argon on reducing the pressure, characteristic for microporous materials (Figure 3, blue curve). The dissimilarity in the adsorption behaviours of nitrogen and argon can be elucidated by their distinct inherent characteristics. Argon, being a non‐polar monatomic molecule, lacks a preferred adsorption orientation, unlike diatomic nitrogen, which possesses a quadrupole moment. It is conceivable that this quadrupole moment acts as a hindrance, potentially impeding the adsorption of nitrogen within the negatively charged framework.

Based on the argon adsorption measurements, the BET (Brunauer‐Emmett‐Teller) area (S_BET_) of ~150 m^2^ g^−1^ was determined using the adsorption data in *p/p_0_* range of 0.03–0.1. The pore volume (*V_p_*) and average pore size were evaluated using the DFT calculations. The pore size distribution is graphically shown as the inset of Figure 3 (black curve). The comparison of the observed BET value with BET areas reported recently for other materials containing H_2_TPPS^4−^ ligand and lanthanide central atom shows, that **UPJS‐17** has a lower surface area that e. g. UPJS‐10 (259 m^2^ g^−1^) and UPJS‐12 (229 m^2^ g^−1^).^[20]^

**Figure 4:**
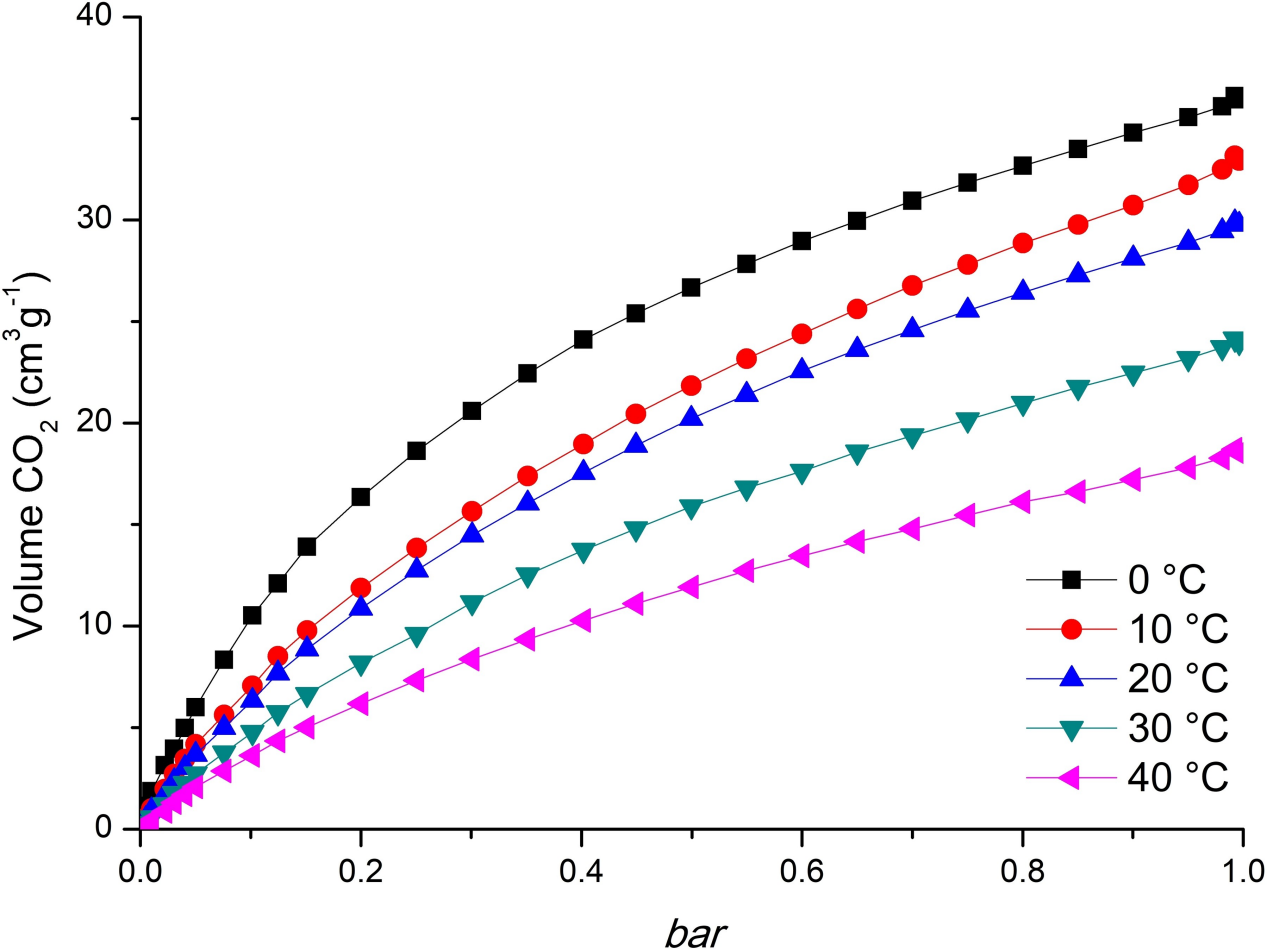
Carbon dioxide isotherms of **UPJS‐17** measured in the temperature range of 0 °C to 40 °C with 10 °C step.

Carbon dioxide adsorption isotherms on activated compound **UPJS‐17** were collected at 0, 10, 20, 30 and 40 °C. The isotherms are shown in Figure 4 and corresponding adsorbed amounts of the carbon dioxide are listed in Table 1. As it was observed, with increasing temperature from 0 to 40 °C, the adsorbed amounts of CO_2_ decrease from 7.0 wt % at 0 °C to 3.2 wt % at 40 °C and 1 bar. Obtained values are moderate compared to the MPFs materials containing same ligand and inner transition ions and could be sorted in following order: La(III) (0.9 mmol)<Eu(III) (1.2 mmol)<Ho(III) (1.6 mmol; **UPJS‐17**)<Sm(III) (1.9 mmol)<Ce(III) (2.0 mmol)<Pr(III) (2.2 mmol).[^20^, ^21^]

**Table 1:** Carbon dioxide adsorption capacities of **UPJS‐17** measured at different temperatures.

CO_2_ uptake at 1 bar			
Temperature	(cm^3^ g^−1^)	(mmol)	(wt %)
0 °C	36.1	1.6	7.0
10 °C	33.2	1.4	6.3
20 °C	29.9	1.2	5.5
30 °C	24.1	0.9	4.3
40 °C	18.7	0.7	3.2

**Figure 5:**
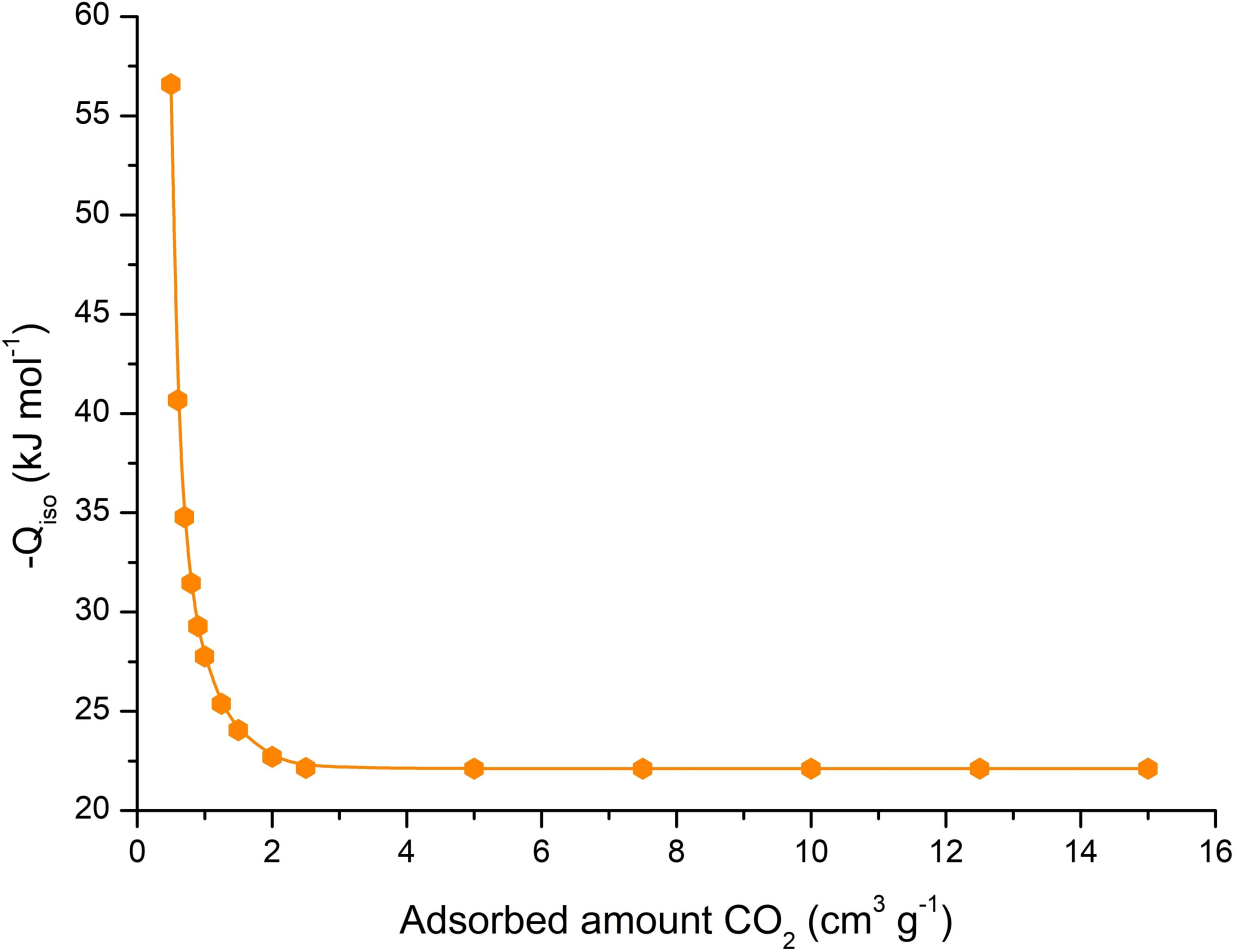
Isosteric heats, Qiso for **UPJS‐17**.

Furthermore, from carbon dioxide adsorption isotherms measured at different temperatures, isosteric heats of CO_2_ adsorption, *Q_iso_* were obtained. *Q_iso_* values were calculated from the slope of adsorption isosteres using the Clausius‐Clapeyron equation: *Q_iso_* =−2.303 *R*[d(log *p*)/d(1/*T*)], where *R* is the universal gas constant, *p* is pressure and *T* is thermodynamic temperature.^[22]^ The values of ‐*Q_iso_* evaluated for **UPJS‐17** (Figure 5). It is obvious from the figure, that isosteric heats exponentially decrease from the beginning of the adsorption. The initial values of the isosteric heats of adsorption, at low CO_2_ loadings, are relatively high, 56.5 kJ mol^−1^, and correspond to the interaction of the carbon dioxide molecules with four uncoordinated nitrogen atoms from tetrapyrrole core of the porphyrin ring.^[23]^ This value is comparable to the isosteric heats observed for carbon dioxide adsorption on amine modified mesoporous silica^[24]^ or amine substituted MOFs.^[25]^ At higher loadings of carbon dioxide the isosteric heats decrease to the values around 22 kJ mol^−1^, which are comparable to the pristine, “non‐amine“ MOF materials.^[26]^ CO_2_ uptake capacities **UPJS‐17** are lower with the comparison of not so far published materials ZJNU‐133 and ZJNU‐14 (63.1 and 28.2 respectively, at 298 K, 1 atm). Conversely, the isosteric heats of CO_2_ adsorption at low loading are notably higher, measuring 21.0, 26.2, and 56.5 kJ mol^−1^ for ZJNU‐140, ZJNU‐133, and UPJS‐17, respectively.[^27^, ^28^]

**Figure 6:**
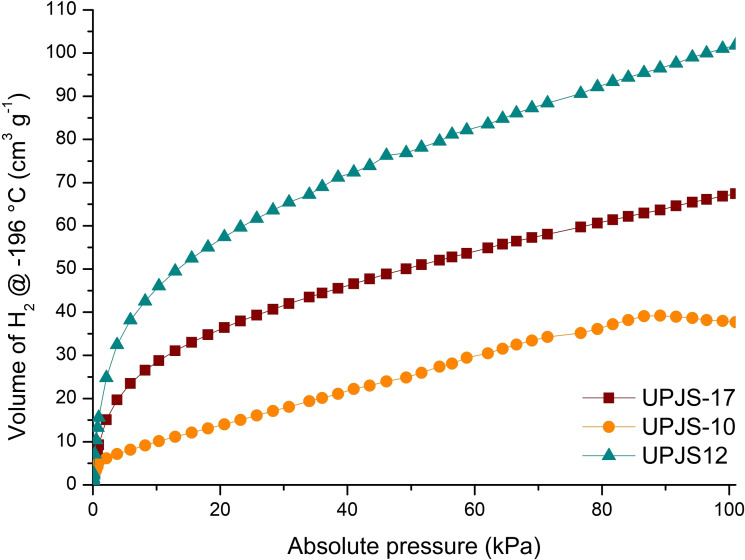
Hydrogen adsorption isotherms at −196 °C of **UPJS‐17**, UPJS‐10 and UPJS‐12.

The hydrogen adsorption isotherms of **UPJS‐17** were measured at the temperature of −196 °C up to 1 bar and are shown in Figure 6. Activated material **UPJS‐17** shows a maximum hydrogen uptake of 67 cm^3^ g^−1^ at 1 bar, which corresponds to 10.6 mmol and 2.1 wt %.

Moreover, we have measured hydrogen adsorption also for the compounds we have investigated recently,^[20]^ namely UPJS‐10 (containing Pr(III) ion) and UPJS‐12 (containing Ce(III) ion). The results are also presented in Figure 6. Their adsorption capacities were 1.2 wt % (39 cm^3^ g^−1^, 6.1 mmol) for UPJS‐10 and 3.2 wt % (102 cm^3^ g^−1^, 16.1 mmol)^[19]^ for UPJS‐12. It is obvious that the highest hydrogen uptake was observed for UPJS‐12. In summary, gas storage capacities of the compounds, which we have studied recently are shown in Table S2 in the Supporting Information.

In addition, we have measured hydrogen adsorption for compounds presented in Figure 6 also at the sublimation temperature of dry ice, i. e., −78.5 °C (Figure S3a in the Supporting Information) and also at 20 °C (Figure S3b in the Supporting Information). We observed the expected rapid decrease in the adsorption of hydrogen with increasing temperature. Compound UPJS‐10 adsorbed 3.9 cm^3^ g^−1^ of dihydrogen at −78.5 °C, which corresponds to 0.24 mmol g^−1^ of this gas. For **UPJS‐17**, we observed slightly higher adsorbed amount, 4.1 cm^3^ g^−1^, which corresponds to 0.25 mmol g^−1^ of hydrogen. The highest adsorbed amount at −78.5 °C was achieved, similarly to the measurements at −196 °C, for the compound UPJS‐12, when this complex adsorbed 8.32 cm^3^ g^−1^ of hydrogen, corresponding to 0.52 mmol g^−1^. Further increase in the temperature to 20 °C led to continuous decrease in adsorption. At 20 °C **UPJS‐17** adsorbed the smallest amount of hydrogen (0.9 cm^3^ g^−1^), followed by UPJS‐10, which adsorbed 1.3 cm^3^ g^−1^. Again, the highest adsorbed value was achieved for UPJS‐12 (1.7 cm^3^ g^−1^). Summarizing the results of dihydrogen adsorption measurements at different temperatures, it can be noted that the best hydrogen adsorption properties were observed for UPJS‐12 which contained Ce(III) ions, then followed by **UPJS‐17** containing Ho(III) ions. The lowest adsorption capacities were observed for UPJS‐10, containing Pr(III) ions.

## Conclusions

In conclusion, the novel holmium‐based porous porphyrin framework, **UPJS‐17** {(H_3_O^+^)[Ho(H_2_TPPS)]^−^ ⋅ 4H_2_O}_n_, has been prepared and investigated. **UPJS‐17** exhibit permanent porosity formed by three mutually crossing cavities propagating along all crystallographic axes. Calculated BET surface area from Ar adsorption isotherms is ~150 m^2^ g^−1^. The CO_2_ adsorption was measured at different temperatures, with the highest uptake of 7 wt % CO_2_ at 0 °C and 1 bar. Hydrogen adsorption at different temperatures (−196 °C, −78.5 °C and 20 °C) was also studied. The isosteric heats of adsorption of CO_2_ were calculated from CO_2_ isotherms measured at different temperatures and Q_iso_ fall in the range of 56.5–24 kJ mol^−1^. To the best of our knowledge, the present work represents the first study of hydrogen adsorption on H_6_TPPS based MPFs. The highest adsorbed amount of hydrogen for **UPJS‐17** was 2.1 wt % of H_2_ at −196 °C and 1 bar. However, higher amount (3.2 wt %) was observed for UPJS‐12, structurally described in our previous study.^[20]^

## Experimental section

### Synthesis of UPJS‐17

Synthesis of {(H_3_O^+^)[Ho(H_2_TPPS)]^−^ ⋅ 4H_2_O}_n_ (**UPJS‐17**): The dark green needles of compound UPJS‐10 were prepared by hydrothermal synthesis. A mixture of H_6_TPPS (50 mg, 0.05 mmol), Ho(NO_3_)_3_ ⋅ 6H_2_O (23.46 mg, 0.05 mmol), and 15 mL of distilled water were loaded into a 45 mL Teflon‐lined stainless steel autoclave and heated to 200 °C with a heating rate of 10 °C min^−1^ and kept at this temperature for 5 days. After this time the autoclave was cooled down with a cooling rate of 5 °C min^−1^ to laboratory temperature. The obtained crystals were washed with acetone three times over a filter. The yield was 20 % (based on Ho). Elemental Anal. Calc. for **UPJS‐17** (C_44_H_37_N_4_O_17_S_4_Ho; Mw=1186.97 g mol^−1^): C, 44.52 %; H, 3.14 %; N, 4.72 %; S, 10.81 %. Found C, 44.38 %; H, 3.18 %; N, 4.72 %; S, 10.91 %. IR (KBr): ν, cm^−1^ 3505 (m), 3319(w), 3095 (m), 1620(w), 1481 (m), 1394 (w), 1251 (s), 1167 (s), 1122 (vs), 1032 (vs), 731 (s) and 624 (s).

### X‐ray structure determination

The single‐crystal X‐ray diffraction data set was measured on a Nonius Kappa CCD diffractometer equipped with a Bruker APEX II detector. For **UPJS‐17** Cu/Kα (λ=1.54178 Å) at 288(2) K radiation was used. Data reduction was carried out by the diffractometer software. The phase problem was solved by direct methods and refined with full‐matrix least‐squares on F^2^ using the Shelx‐18 program suite.^[29]^ Hydrogen atoms were refined isotropically and all other atoms anisotropically. Hydrogen atoms residing on aromatic carbon atoms were included in an ideal position with the C−H bond fixed to 0.95 Å and Uiso(H) assigned to 1.2 U_eq_ of the adjacent carbon atom. The contribution of guest molecules located in the pores was substracted by the SQUEEZE procedure in Platon.^[18]^ The structure figures were drawn using DIAMOND software.^[30]^ Crystal data for **UPJS‐17** is summarized in Table S1 in the Supporting Information.

Deposition Number 2167706 (for **UPJS‐17**) contains the supplementary crystallographic data for this paper. These data are provided free of charge by the joint Cambridge Crystallographic Data Centre and Fachinformationszentrum Karlsruhe Access Structures service.

### Characterization

Elemental analysis was performed using an CHNOS Elemental Analyzer vario MICRO from Elementar Analysensysteme GmbH.

Powder X‐ray diffraction (PXRD) experiments were done in reflection geometry using a Rigaku Ultima IV multipurpose diffractometer. In order to achieve parallel and clean X‐ray beam for PXRD experiments, initially divergent Cu/Kα radiation (*λ*=1.54056 Å) emitted from X‐ray lamp was further guided through the multi‐layered mirror and set of slits. Powder samples were loaded in metal frame PXRD experiments were done by 2*θ* continuous scan at 0.5° min^−1^ from 5° to 50° and diffracted photons were recorded using a NaI scintillation detector.

Diffuse reflectance infrared Fourier transform (DRIFT) measurements were performed at the Nicolet Avatar FT‐IR 6700 spectrometer, equipped with an electromagnetic source in the mid‐infrared region (4000–500 cm^−1^) and a DTGS detector. For each spectrum, 200 scans were accumulated with a resolution of 4 cm^−1^. IR measurements were performed by the Praying Mantis in‐situ cell under vacuum and the pure KBr was measured as a reference background.

Thermogravimetry (TGA) and differential thermal analysis (DTA) were used to study the thermal behaviour in the temperature range 25–900 °C, with a heating rate of 6 °C min^−1^ using a STA Netzsch 409PC instrument.

Adsorption measurements for various gases (Ar, N_2_, CO_2_ and H_2_) were preceded by degassing of all samples at 150 °C for 16 hours under dynamic vacuum. The surface areas and pore volumes of the samples were measured by argon sorption at −186 °C (by nitrogen sorption at −196 °C) using a Quantachrome AUTOSORB‐iQ‐C automated gas sorption system. The total surface area was calculated via the Brunauer‐Emmett‐Teller (BET) equation and the micropore volume was obtained by means of the DFT (NLDFT kernel) method. Adsorption isotherms of carbon dioxide at various temperatures were measured using a Quantachrome AUTOSORB‐iQ‐C with a combined volumetric and dynamic sorption system. The hydrogen (99.999 % purity) adsorption measurements were measured on the static manometric adsorption system, Autosorb iQ‐XR developed by Quantachrome Instruments. The H_2_ isotherms were measured in the range of 0.5–101 kPa of absolute pressure.

## Supporting Information

The authors have cited additional references within the Supporting Information (Ref. [31–41]).

## Author Contributions

Nikolas Király – conceptualization, data curation, formal analysis, investigation, validation, visualization, writing – original draft. Vladimír Zeleňák – funding acquisition, methodology, project administration, supervision, writing – review & editing. Tomáš Zelenka – data curation, validation. Miroslav Almáši – data curation, validation. Juraj Kuchár – data curation, validation.

## Conflict of interest

The authors declare no conflict of interest.

1

## Supporting information

As a service to our authors and readers, this journal provides supporting information supplied by the authors. Such materials are peer reviewed and may be re‐organized for online delivery, but are not copy‐edited or typeset. Technical support issues arising from supporting information (other than missing files) should be addressed to the authors.

Supporting Information

## Data Availability

The data that support the findings of this study are available in the supplementary material of this article.
