# Supplementary material for: A New Member of the Metal‐Porphyrin Frameworks Family: Structure, Physicochemical Properties, Hydrogen and Carbon Dioxide Adsorption
Source: ChemistryOpen. 2023 Nov 9;13(2):e202300100. doi: 10.1002/open.202300100 (PMC10853072; doi:10.1002/open.202300100)
Supplement: Supplementary file 1 — Supporting Information [file OPEN-13-e202300100-s001.pdf]

ChemistryOpen

Supporting Information

A New Member of the Metal-Porphyrin Frameworks Family: Structure, Physicochemical Properties, Hydrogen and Carbon Dioxide Adsorption

Nikolas Király, Vladimír Zeleňák,* Tomáš Zelenka, Miroslav Almáši, and Juraj Kuchár

Materials.

All chemicals were obtained from commercial sources, Sigma-Aldrich or PorphyChem, and used without further purification. Meso-tetraphenylporphirin-4,4′,4″,4‴-tetrasulfonic acid (PorphyChem, 98%), $Ho(NO_3)_3{\cdot}6H_2O$ (Sigma-Aldrich, 99,999%).

Synthesis of UPJS-17:

Synthesis of $\{(H_3O^+)[Ho(H_2TPPS)]^-{\cdot}4H_2O\}_n$ (**UPJS-17**): The dark green needles of compound UPJS-10 were prepared by hydrothermal synthesis. A mixture of meso-tetraphenylporphirin-4,4′,4″,4‴-tetrasulfonic acid H_6TPPS (50mg, 0.05 mmol), $Ho(NO_3)_3{\cdot}6H_2O$ (23.46 mg, 0.05 mmol), and 15 ml of distilled water were loaded into a 45 ml Teflon-lined stainless steel autoclave and heated to 200 °C with a heating rate of 10 °C min^{-1} and kept at this temperature for 5 days. After this time the autoclave was cooled down with a cooling rate of 5 °C min^{-1} to laboratory temperature. The obtained crystals were washed with acetone three times over a filter. The yield was 20 % (based on Ho). Elemental Anal. Calc. for **UPJS-17** ($C_{44}H_{37}N_4O_{17}S_4Ho$; Mw = 1186.97 g mol^{-1}): C, 44.52%; H, 3.14%; N, 4.72%; S, 10.81%. Found C, 44.38%; H, 3.18%; N, 4.72%; S, 10.91%. IR (KBr): ν, cm^{-1} 3505 (m), 3319(w), 3095 (m), 1620(w), 1481 (m), 1394 (w), 1251 (s), 1167 (s), 1122 (vs), 1032 (vs), 731 (s) and 624 (s).

X-ray structure determination

The single-crystal X-ray diffraction data set was measured on a Nonius Kappa CCD diffractometer equipped with a Bruker APEX II detector. For **UPJS-17** Cu/Kα (λ = 1.54178 Å) at 288(2) K radiation was used. Data reduction was carried out by the diffractometer software. The phase problem was solved by direct methods and refined with full-matrix least-squares on F^2 using the Shelx-18 program suite.[31] Hydrogen atoms were refined isotropically and all other atoms anisotropically. Hydrogen atoms residing on aromatic carbon atoms were included in an ideal position with the C−H bond fixed to 0.95 Å and Uiso(H) assigned to 1.2Ueq of the adjacent carbon atom. The contribution of guest molecules located in the pores was substracted by the SQUEEZE procedure in Platon.[32] The structure figures were drawn using DIAMOND software.[33] Crystal data for **UPJS-17** is summarized in Tab. S1 in ESI. Crystal structures of **UPJS-17** is deposited with CSD (2167706) and can be retrieved free of charge from https://www.ccdc.cam.ac.uk/structures.

Table S1 Crystal data for **UPJS-17**.

Identification code	**UPJS-17**
Empirical formula	$C_{44}H_{24}N_4O_{12}S_4Ho$
Formula weight	1093.84
Temperature	288(2) K
Wavelength	1.54184 Å
Crystal system	Tetragonal
Space group	P4/mcc
Unit cell dimensions	a =15.3829(9) Å
	b = 15.3829(9) Å
	c =9.7787(15) Å
Volume	2314.0(4) $Å^3$
Z	2
Density (calculated)	1.570 mg/m^3
Absorption coefficient	5.436 mm^{-1}
F(000)	1086
Crystal size	0.080 x 0.058 x 0.035 mm^3
Theta range for data collection	2.873 to 74.938°.
Index ranges	-11<=h<=19,<br>-18<=k<=11,<br>-11<=l<=10
Reflections collected	4189
Independent reflections	1241[R(int) = 0.0398]
Data completeness to theta = 67.684°	100.0%
Refinement method	Full-matrix least-squares on F^2
Data / restraints / parameters	1526 / 6 / 94
Goodness-of-fit on F^2	1.186
Final R indices [I>2sigma(I)]	R1 = 0.0625, wR2 = 0.2025
R indices (all data)	R1 = 0.0716, wR2 = 0.2146
Extinction coefficient	n/a
Largest diff. peak and hole	1.189 and -0.729 e.$Å^{-3}$
Deposition Number	2167706

Characterization

The elemental analysis was performed using an CHNOS Elemental Analyzer vario MICRO from Elementar Analysensysteme GmbH.

Powder X-ray diffraction (PXRD) experiments were done in reflection Bragg-Brentano geometry using a Bruker D2 Phaser diffractometer. Powder samples were loaded in metal frame PXRD experiments were done by 2θ continuous scan at 0.5° min^{-1} from 5° to 35° and diffracted photons were recorded using a SSD160 detector.

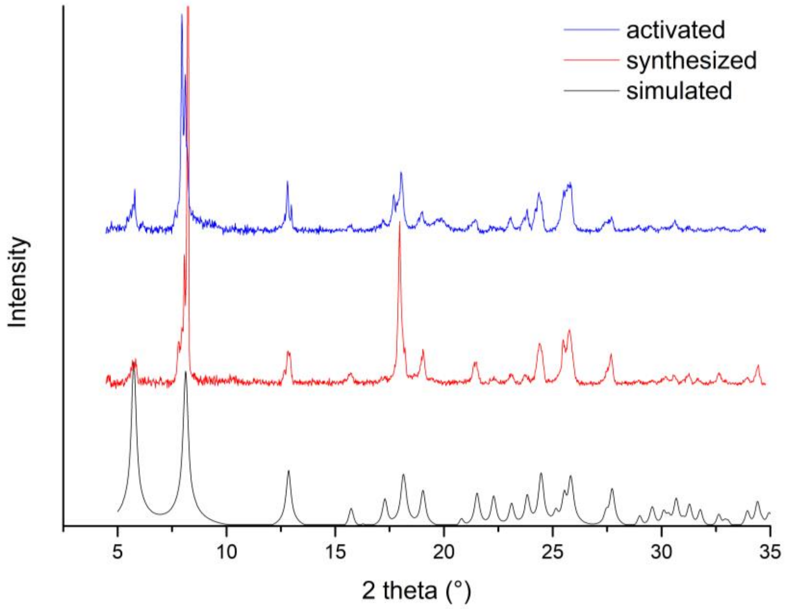

Figure S1 Comparison of PXRD patterns of measured **UPJS-17** (red curve), calculated **UPJS-17** data from SXRD measurements (black curve) and sample after activation and adsorption measurements (blue curve).

The diffuse reflectance infrared Fourier transform (DRIFT) measurements were performed at the Nicolet Avatar FT-IR 6700 spectrometer, equipped with an electromagnetic source in the mid-infrared region (4000 – 500 cm^{-1}) and a DTGS detector. For each spectrum, 200 scans were accumulated with a resolution of 4 cm^{-1}. IR measurements were performed by the Praying Mantis in-situ cell under vacuum and the pure KBr was measured as a reference background.

The thermal behaviour was studied by thermogravimetry (TGA) combined with differential thermal analysis (DTA) in the temperature range 25-900 °C, with a heating rate of 6 °C min^{-1} using a STA Netzsch 409PC instrument.

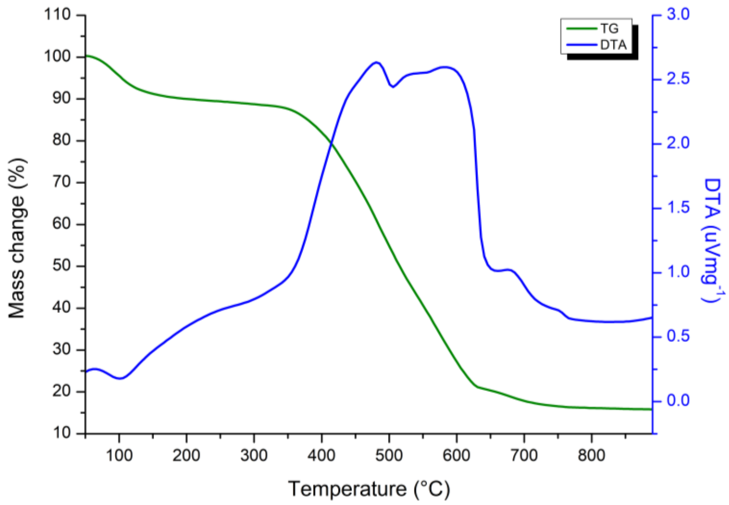

Figure S2 The results of TG/DTA characterization for **UPJS-17** sample.

Before all adsorption measurements of various gases (Ar, N_2, CO_2 and H_2), samples were degassed at 150 °C for 16 hours under dynamic vacuum. The surface areas and pore volumes of the samples were measured by argon sorption at -186 °C (by nitrogen sorption at -196 °C) using a Quantachrome AUTOSORB-iQ-C automated gas sorption system. The total surface area was calculated via the Brunauer Emmett Teller (BET) equation and the micropore volume was obtained by means of the DFT (NLDFT kernel) method. Adsorption isotherms of carbon dioxide at various temperatures were measured using a Quantachrome AUTOSORB-iQ-C with a combined volumetric and dynamic sorption system. The hydrogen (99.999% purity) adsorption measurements were measured on the static manometric adsorption system, Autosorb iQ-XR developed by Quantachrome Instruments. The H_2 isotherms were measured in the range of 0.5–101 kPa of absolute pressure.

Table S2 Gas storage capacities of compounds with UPJS acronym.

**Composition**	**Acronym**	***N_2 @ -196 °C***	***Ar @ -186 °C***	***H_2 @ -196°C***	***CO_2***	***T***	***Ref***
		S_{BET}	S_{BET}	*1 atm*	*1 atm*		
		[m^2 g^{-1}]	[m^2 g^{-1}]	*wt. %*	*wt. %*	[°C]	
{[$Sr_4(O)(L1)_{3/2}(H_2O)_4$]·4DMF·4$H_2O$}$_n$	**UPJS-8**	-	-	2.17	2.64	20	34
{[$Ca_4(O)(L1)_{3/2}(H_2O)_4$]·4DMF·4$H_2O$}$_n$	**UPJS-7**	103	126	3.65	3.92	20	34
{[$Zn_2(L1)(H_2O)_2$]·3DMF·3H_2O}$_n$	**UPJS-2**	248	-	-	5.67<br>4.74	0<br>20	35
{[$Ba_3(L1)_{3/2}(H_2O)_6$]·2DMF·4H_2O}$_n$	**UPJS-9**	320	358	1.8	2.41	20	34
{[$Zn_2(L1)(L8)_2$]·2DMF·7H_2O}$_n$	**UPJS-4**	-	644	1.28	10.5<br>7	0<br>20	36
{[$Ni_4(L1)_2(H_2O)_8$]·10DMF·11H_2O}$_n$	**UPJS-3**	700	-	-	12.4<br>6.5	0<br>20	37
{[$Pb_4(L1)_2(H_2O)_4$]·5DMF·H_2O}$_n$	**UPJS-5**	980	-	-	9.3	0	38,39
{[$Cd_2(L2)$]·5H_2O·4DMF}$_n$	**UPJS-14**	830	-	-	3.52	30	40
{[$Zn_2(L2)$]·4H_2O·3DMF}$_n$	**UPJS-13**	1057	-	-	4.14	30	40
{[$Pr_4(H_2TPPS)_3$]·11H_2O}$_n$	**UPJS-10**	**-**	259	1.2	9.83<br>6.95	0<br>20	41, TP
{[$Ce_4(H_2TPPS)_3$]·11H_2O}$_n$	**UPJS-12**	**-**	229	3.2	8.58<br>5.99	0<br>20	41, TP
{[$Ho(H_2TPPS)$]·H_3O^+·4H_2O}$_n$	**UPJS-17**	-	153	2.1	7.01<br>5.55	0<br>20	TP

L1 = methanetetrabenzoic acid, *L2* = 4,4',4'',4'''-(4,4',4'',4'''-methanetetrayltetrakis(benzene-4,1-diyl)tetrakis(aza))tetrakis(methan-1-yl-1-yliden)tetrabenzoic acid, H_6TPPS = meso-tetraphenylporphine-4,4′,4″,4‴-tetrasulfonic acid, DMF = *N,N´*-dimethylformamide, TP = this publication.

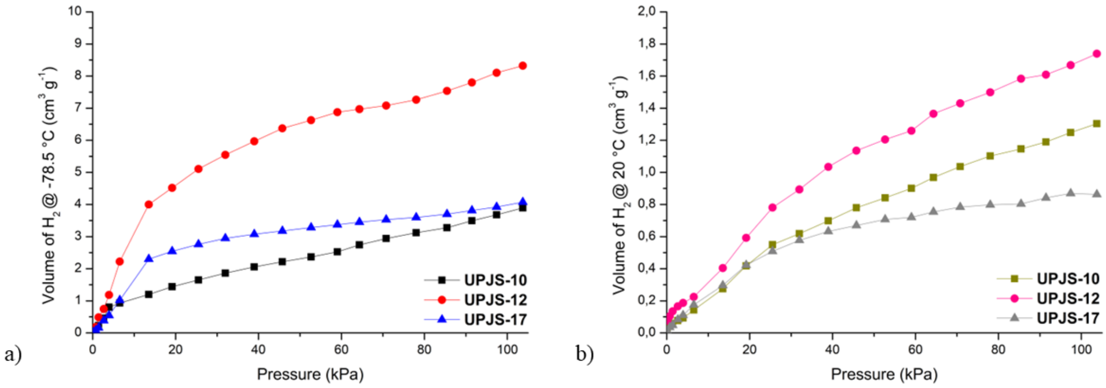

Figure S3 The hydrogen adsorption isotherms of **UPJS-17** compared with isotherms of UPJS-10 and UPJS-12 measured at a) -78.5 °C and b) 20 °C.

References:

[31] G. M. Sheldrick, *Acta Crystallogr., Sect. C: Struct. Chem.* **2015**, *71*, 3−8.

[32] A. L. Spek, *Acta Crystallogr., Sect. D: Struct. Biol.* **2009**, *65*, 148−155.

[33] K. Brandenburg, Crystal Impact GbR: Bonn, Germany **2010**

[34] M. Almáši, V. Zeleňák, R. Gyepes, Ľ. Zauška, S. Bourrelly, *RSC Adv.* **2020**, *10*, 32323–32334.

[35] M. Almáši, V. Zeleňák, R. Gyepes, A. Zukal, J. Čejka, *Colloids Surf., A* **2013**, *437*, 101–107.

[36] M. Almáši, V. Zeleňák, A. Zukal, J. Kuchár, J. Čejka, *Dalton Trans.* **2016**, *45*, 1233–1242.

[37] M. Almáši, V. Zeleňák, M. Opanasenko and J. Čejka, *Dalton Trans.* **2014**, *43*, 3730.

[38] M. Almáši, V. Zeleňák, R. Gyepes, S. Bourrelly, M. V. Opanasenko, P. L. Llewellyn, J. Čejka, *Inorg. Chem.* **2018**, *57*, 1774–1786.

[39] M. Almáši, V. Zeleňák, M. V. Opanasenko, J. Čejka, *Catal. Lett.* **2018**, *148*, 2263–2273.

[40] M. Almáši, N. Király, V. Zeleňák, M. Vilková, S. Bourrelly, *RSC Adv.* **2021**, *11*, 20137–20150.

[41] N. Király, V. Zeleňák, N. Lenártová, A. Zeleňáková, E. Čižmár, M. Almáši, V. Meynen, A. Hovan, R. Gyepes, *ACS Omega* **2021**, *6*, 24637–24649
